# Progress in the application of epimedium and its major bioactive components in the treatment of orthopedic diseases

**DOI:** 10.3389/fphar.2025.1628602

**Published:** 2025-08-12

**Authors:** Dan Tong, Long Chen, Zeyi Jiang, Xuxia Ye, Mengjie Ma, Angzhi Ye, Jian Xu

**Affiliations:** ^1^ Department of Clinical Laboratory, Affiliated Hospital of Shaoxing University, Shaoxing, China; ^2^ School of Medical Technology and Information Engineering, Zhejiang Chinese Medical University, Hangzhou, China; ^3^ Department of Clinical Laboratory, Traditional Chinese Medical Hospital of Zhuji, Zhuji, Zhejiang, China

**Keywords:** epimedium, orthopedic diseases, target, enrichment analysis, pathway

## Abstract

Epimedium brevicornu (Yin Yang Huo), a widely used traditional Chinese medicinal ingredient, has garnered significant attention for its role in treating orthopedic diseases such as osteoporosis. Our work through network pharmacology and bioinformatics analysis, we identified that out of 27 major active components in Epimedium brevicornu, 8 key components have therapeutic effects on 11 types of diseases related to orthopedic conditions. The disease-target association analysis indicated that Osteoarthritis, Osteoporosis, Muscle Spasm and Myopathy have relatively clear targets for disease treatment. The KEGG enrichment analysis results indicate that the signaling pathway of Epimedium treatment in Osteoarthritis may be closely related to the Lipid and atherosclerosis pathway, PPAR signaling pathway and Arachidonic acid metabolism. Epimedium may treat osteoporosis with Nitrogen metabolism, GABAergic synapse, and Pathways in cancer. Epimedium may affect muscle spasticity through Neuroactive ligand-receptor interaction, Serotonergic synapse and Cholinergic synapse closely related to nervous system function; Additionally, our analysis suggests that Epimedium may treat myopathy through Nitrogen metabolism and GABAergic synapse pathways. These studies have not only provided a molecular mechanism-based explanation for the pharmacological effects of Epimedium, but also laid a theoretical foundation for the development of Epimedium-based precision therapeutic regimens.

## 1 Introduction

**FIGURE 1:**
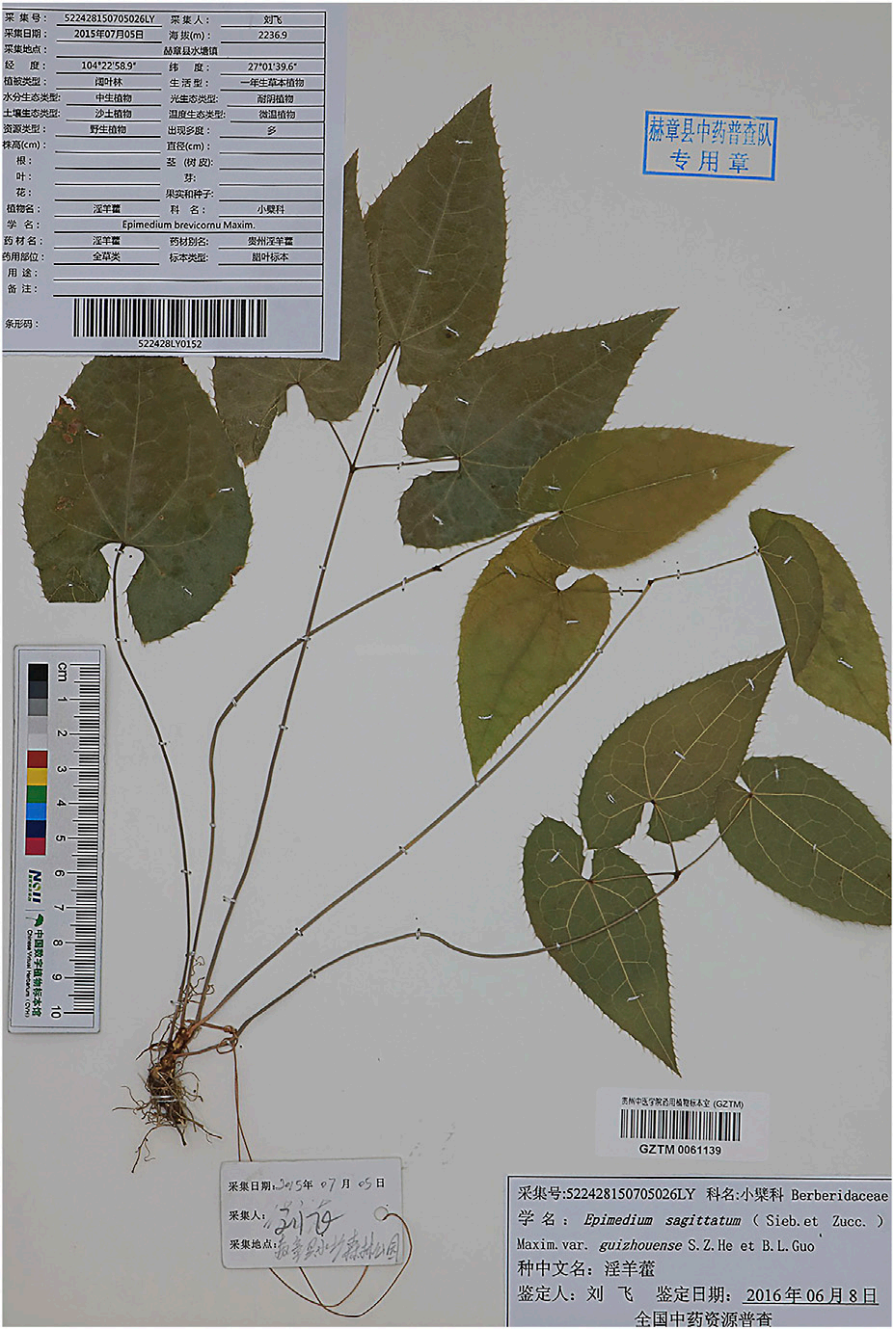
Image of Epimedium brevicornu (Photo source: China Digital Herbarium, CVH).

**FIGURE 2:**
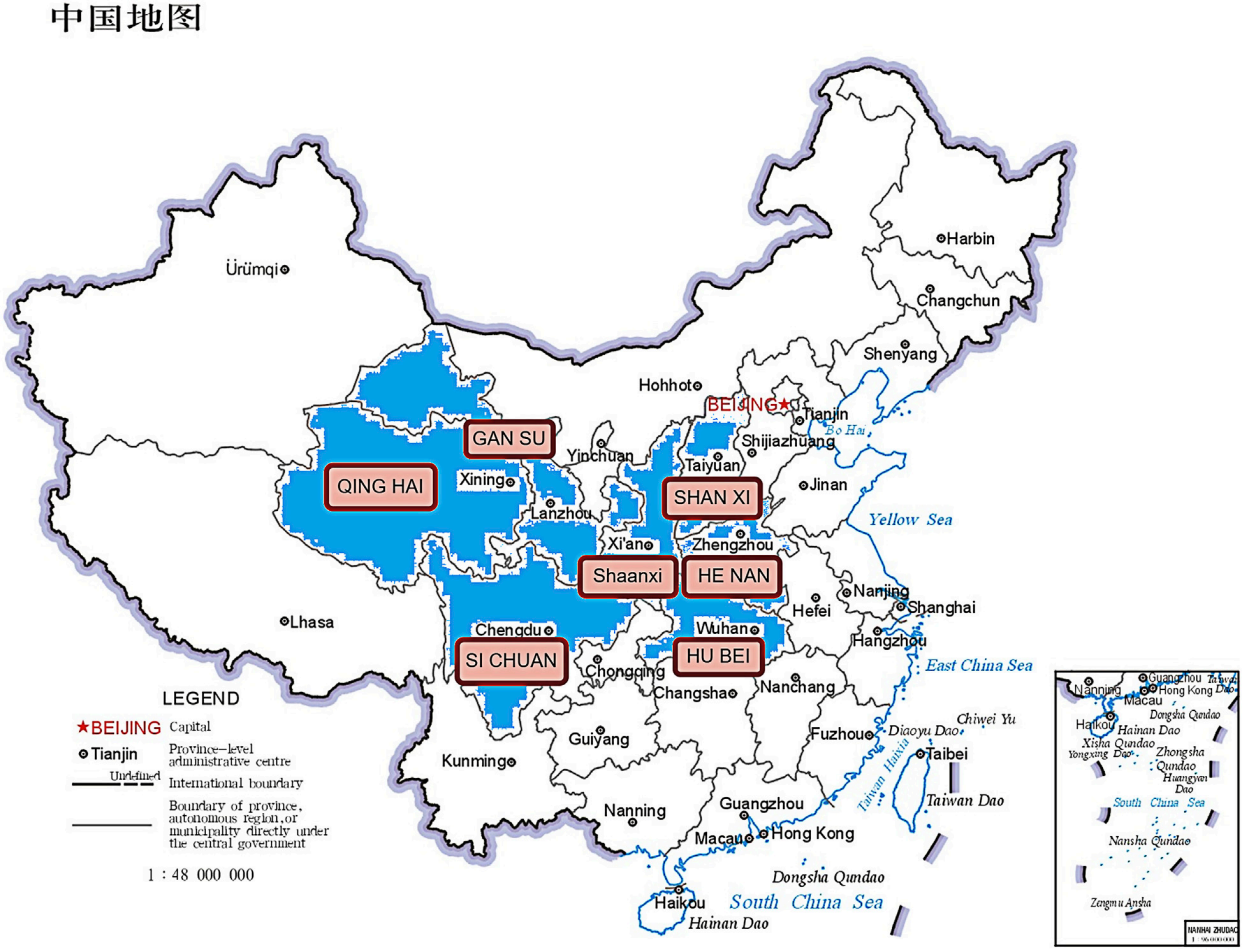
The wild distribution of Epimedium shown in the, ETCM database in China.

Epimedium brevicornu (Figure 1), is a genus of about 52 species in the family Berberidaceae, about 80% of which are endemic to China (Chen et al., 2015). According to the Chinese Pharmacopoeia (2020 edition), it includes the dry leaves of Epimedium brevicornu Maxim, Epimedium sagittatum (Sieb. et Zucc.) Maxim, Epimedium pubescens Maxim and Epimedium koreanum Nakai. The four species of Epimedium recorded in the Chinese Pharmacopoeia have similar pharmacological effects but differ in their geographical distribution. According to The Encyclopedia of Traditional Chinese Medicine (ETCM), the wild distribution of Epimedium in China is primarily concentrated in the central region (Figure 2).

Epimedium was first recorded in Shen Nong Ben Cao Jing (Han Dynasty of China), and has been utilized for treating diseases for approximately 2000 years. It is characterized by its warm nature and bitter taste, and is associated with the liver and kidney meridians. According to the Yet another Traditional Chinese Medicine (YaTCM) database, Epimedium has the effects of reinforcing the kidney yang, strengthening the tendons and bones, and relieving rheumatic conditions, so it is used to treat impotence, seminal emission, weakness of the limbs, rheumatoid arthralgia with numbness and muscle contracture, and climacteric hypertension. In both China and Japan, Epimedium is widely used, either alone or in formulations, for the treatment of orthopedic diseases (Xie et al., 2005; Wang L. et al., 2016).

Recent studies have revealed that the pharmacological effects of Epimedium have transcended its traditional orthopedic applications and regional usage limitations. Its active component icariin has been demonstrated to exert multi-system regulatory effects: In Reproductive System, by inhibiting the NLRP3 inflammasome, icariin significantly ameliorates pyroptosis of Leydig cells and insulin resistance in obese mice, thereby alleviating spermatogenic dysfunction (Wei et al., 2025). In Nervous System, through upregulating the HRD1-mediated ubiquitination pathway, it promotes AβPP degradation, consequently improving cognitive function in APP/PS1 mice (Chen et al., 2025). In Musculoskeletal System, through synergistic downregulation of inflammatory factors IL-1β, IL-6, TNF-α, and MMP-9, icariin induces apoptosis of fibroblast-like synoviocytes in rheumatoid arthritis while suppressing their invasive metastasis, demonstrating remarkable anti-arthritic activity (Ding et al., 2025). These advancements not only expand the clinical potential of Epimedium but also provide valuable insights for global drug development.

## 2 The main biological components and chemical structures of epimedium

The plant chemistry research of Epimedium genus began in 1935 (Akai, 1935). The researchers have detected more than 260 components from Epimedium, including 141 flavonoids, 31 lignins, and many other types of compounds (Ma et al., 2011). As the main organ of plants, leaves have effects on plant development and biomass, and are the main medicinal site in traditional Epimedium herbs (Yu et al., 2023). Some scholars believe that flavonoids in Epimedium leaves are the most important and significant active ingredients in Epimedium (Wu et al., 2003; Pei and Guo, 2007). According to the Integrative Pharmacology-based Research Platform of Traditional Chinese Medicine (TCMIP) database, Epimedium contains 27 main biological components, whose chemical structures and molecular formulas are listed in Table 1.

**TABLE 1:** Main biological components and chemical structures of Epimedium.

Chemical component	Chemical structure	Molecular formula	Chemical component	Chemical structure	Molecular formula
Cetylic Acid, Hexadecanoic Acid, Palmitic Acid	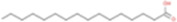	C16H32O2	Anhydroicaritin-3-O-Î‘-L-Rhamnosyl-7-O-Î’-D-Glucopyranoside	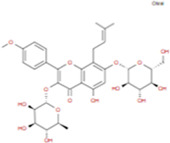	C33H40O15
Linolenic Acid	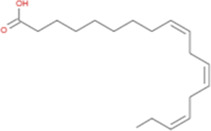	C18H30O2	Baohuoside Ii	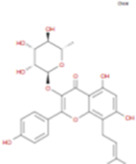	C26H28O10
Octadecanoic Acid, Stearic Acid	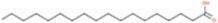	C18H36O2	Hyperin, Hyperoside,Hyperoside,Quercetin-3-O-Galactoside	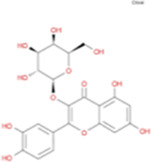	C21H20O12
Magnoflorine	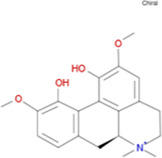	C20H24NO4	Bilobanol	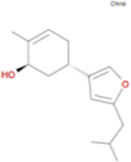	C15H22O2
Kaempferitrin	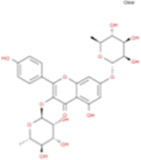	C27H30O14	Breviflavone B	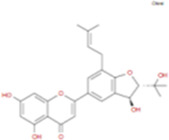	C25H26O7
Hentriacontane	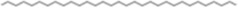	C31H64	Des-O-Methylicariin	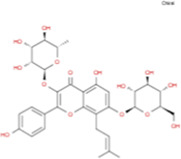	C32H38O15
Diphylloside A	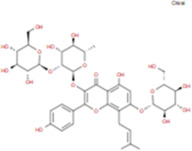	C38H48O20	Neoicariin	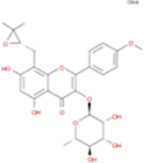	C27H30O11
Epimedin A	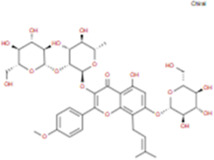	C39H50O20	Wushanicariin	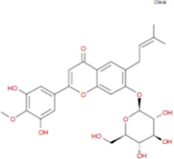	C27H30O11
Epimedin B	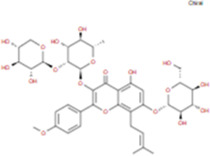	C38H48O19	Yinyanghuo A	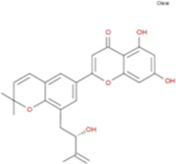	C25H24O6
Epimedin C	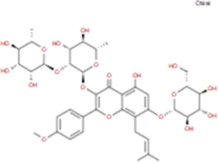	C39H50O19	Yinyanghuo B	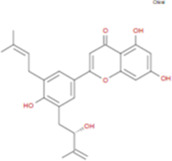	C25H26O6
Epimedokoreanoside I	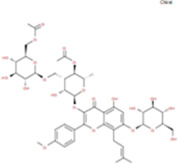	C44H56O22	Yinyanghuo C	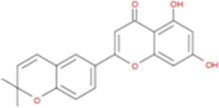	C20H16O5
Epimedokoreanoside Ii	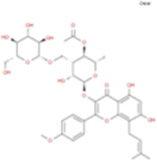	C36H44O16	Yinyanghuo D	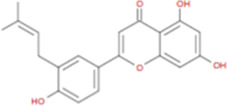	C20H18O5
Epimedoside C	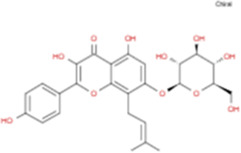	C26H28O11	Yinyanghuo E	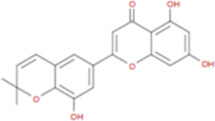	C20H16O6
Ikarisoside F	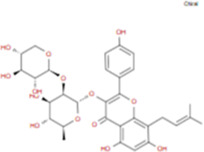	C31H36O14			

In this study, we first retrieved and extracted the main active components of Epimedium and their potential therapeutic effects on bone-related diseases from the TCMIP database (v2.0). Subsequently, using the disease-target association analysis module built into the database, disease-related keywords were employed to screen potential therapeutic targets associated with these conditions. Through network pharmacology, a comprehensive analysis was conducted to explore the potential pharmacological mechanisms and pathways by which Epimedium regulates bone/muscle metabolism, repair, and related diseases.

## 3 The role of the main bioactive substances of epimedium in orthopedic diseases

Epimedium encompasses a diverse array of chemical substances, primarily including Fatty Acids, Flavonoid Glycosides, Alkaloids, Terpenoids and their derivatives, Alkanes, and Other Glycosides. These chemical entities exert various biological functions, and the bioactive functions of each category are detailed in Table 2.

**TABLE 2:** Classification and main functions of chemical substances in Epimedium.

Category	Representative compounds	Primary functions
Fatty Acids	Palmitic Acid	Energy storage, cell membrane structure (Zahradka et al., 2017; Jaureguiberry et al., 2014; Zhao et al., 2023; Balla et al., 2019)
Flavonoid Glycosides	Epimedin A/B/C	Antioxidant, hormone regulation, anti-osteoporosis (Wu et al., 2012; Buranasudja et al., 2022; Yuan et al., 2023; Ammar et al., 2016; Menghini et al., 2016)
Alkaloids	Magnoflorine	Anti-inflammatory, neuroprotection (Kushida et al., 2021; Qu et al., 2013; Luo et al., 2024; Lee et al., 2019; Geetha and Ramachandran, 2021)
Alkanes	Hentriacontane	Plant cuticle protection (Wu et al., 2019)
Terpenoids	Bilobanol	Anti-inflammatory or neuroactive (Ge et al., 2022; Xu et al., 2024; Kabir et al., 2021)

Extensive studies have demonstrated that the characteristic flavonoid glycosides Epimedin A/B/C from Epimedium can be metabolically converted into icariin (also a flavonoid glycoside) both *in vivo* and *in vitro* (Su et al., 2023; Zhou et al., 2015). Icariin promotes osteogenesis through multiple molecular mechanisms:By activating the Wnt/-catenin signaling pathway, it enhances osteoblast proliferation and differentiation, thereby increasing bone density and improving skeletal function (Wei Q. et al., 2016). Meanwhile, it promotes bone formation by stimulating the bone morphogenetic protein (BMP) signaling pathway (Liang et al., 2012). Furthermore, existing evidence indicates that icariin can also suppress osteoclast differentiation by inhibiting the RANKL/NF-κB signaling cascade, thereby reducing bone resorption and preventing bone loss (Kim et al., 2018).

Research has found that the use of alkaloid Magnoflorine in Epimedium can significantly reduce joint swelling and bone erosion. This may be related to its ability to inhibit the production of inflammatory factors and reduce oxidative stress (Liu et al., 2020). Moreover, Magnoflorine may also protect the joints and bones by regulating immune responses and inhibiting osteoblast hyperactivation (Maeda et al., 2022). Further research indicates that Magnoflorine likely exerts its anti-inflammatory effects by influencing the nuclear factor kappa B(NF-κB) signaling pathway. NF-κB is a transcription factor that plays a crucial role in inflammatory responses, and its activation is closely associated with inflammation and bone destruction in rheumatoid arthritis (RA) (Moqbel et al., 2020). By inhibiting the activation of NF - κB, Magnoflorine can reduce the release of inflammatory factors, thereby alleviating joint inflammation and bone loss.

Bilobanol is a natural compound and some scholars have suggested that it may inhibit osteoclast activity by regulating the OPG/RANKL ratio. Hyperactivation of osteoclasts is one of the main causes of bone loss diseases such as osteoporosis. Osteoprotegerin (OPG) and Receptor activator of nuclear factor-κB ligand (RANKL) play crucial roles in the formation and activation of osteoclasts. Research has shown that increasing the expression of OPG or reducing the expression of RANKL can effectively inhibit osteoclast differentiation and function, thereby reducing bone resorption (Iolasc et al., 2011; Sun et al., 2016; Shao et al., 2019). Research has also found that in osteoblasts, fatty acid metabolism and storage are essential for the bone formation process. Osteoblasts can release endogenous fatty acids from lipid droplets through lipolysis to support the cellular bioenergy state and bone formation (Nandy et al., 2023).

In summary, the various components contained in Epimedium may exhibit a comprehensive effect of promoting bone formation, inhibiting bone resorption, and providing anti-inflammatory and analgesic effects through multi-target and multi-pathway synergistic actions.

The research group utilized the TCMIP database to screen the identified chemical components in Epimedium and their potential therapeutic effects on orthopedic diseases, further verifying the correlation between Epimedium and orthopedic diseases. Analysis revealed that Epimedium may have therapeutic potential for 11 types of orthopedic conditions, including Osteoarthritis (OA), Osteoporosis, Abnormality of the Musculature, Bone Cyst, Skeletal Muscle Atrophy, Muscle Spasm, Myopathy, Bone Pain, Osteomyelitis, Osteochondrosis, and Limb Muscle Weakness (Table 3).

**TABLE 3:** Major active components isolated from Epimedium and their potential therapeutic effects on Orthopedic Diseases.

Ingredient Name	Potential Treatable skeletal disorders	Ingredient Name	Potential Treatable skeletal disorders
Cetylic Acid, Hexadecanoic Acid, Palmitic Acid	Osteoarthritis (Sekar et al., 2020; Zhou et al., 2013)	Hyperin, Hyperoside,Hyperoside,Quercetin-3-O-Galactoside	Skeletal Muscle Atrophy
	Osteoporosis (Fillmore et al., 2015)		Osteomyelitis (Huang et al., 2021)
	Abnormality Of The Musculature (Laurentius et al., 2019)		Osteoporosis (Li et al., 2019a; Li et al., 2020; Zhang et al., 2014a)
	Bone Cyst		Muscle Spasm (Zhu et al., 2022)
Linolenic Acid	Osteoarthritis (Harasymowicz et al., 2019; Ioan-Facsinay and Kloppenburg, 2018; Gao et al., 2020)	Epimedoside C	Muscle Spasm (Zagorchev et al., 2016; Wan et al., 2020)
	Osteoporosis (Anand and Kaithwas, 2014; de Abreu et al., 2022)		Skeletal Muscle Atrophy (Lim et al., 2018)
	Skeletal Muscle Atrophy (Wu et al., 2024)		Osteoporosis (Chen et al., 2022)
	Abnormality Of The Musculature (Takić et al., 2024)		Limb Muscle Weakness (Xu et al., 2021)
Magnoflorine	Muscle Spasm (Okon et al., 2020; Slavova-Kazakova et al., 2021)	Octadecanoic Acid, Stearic Acid	Osteoarthritis (Dai et al., 2021)
			Osteoporosis (Seidel et al., 2024)
Kaempferitrin	Myopathy (Wang and Zhao, 2019; Li et al., 2024)		Abnormality Of The Musculature
	Bone Pain (Wong et al., 2019; Alrumaihi et al., 2024)		Bone Cyst (Bastiaansen-Jenniskens et al., 2013)
	Osteomyelitis	Des-O-Methylicariin	Osteoporosis (Chen et al., 2024; Liu et al., 2024a; Ying et al., 2020; Zhang et al., 2014b)
	Osteochondrosis (Cazarolli et al., 2013)		Muscle Spasm (Li et al., 2019b)
	Osteoporosis (Tsuchiya et al., 2018)		Skeletal Muscle Atrophy (Cho et al., 2018; Yi et al., 2022)

## 4 Possible therapeutic targets and mechanisms of epimedium in the treatment of orthopedic diseases

**FIGURE 3:**
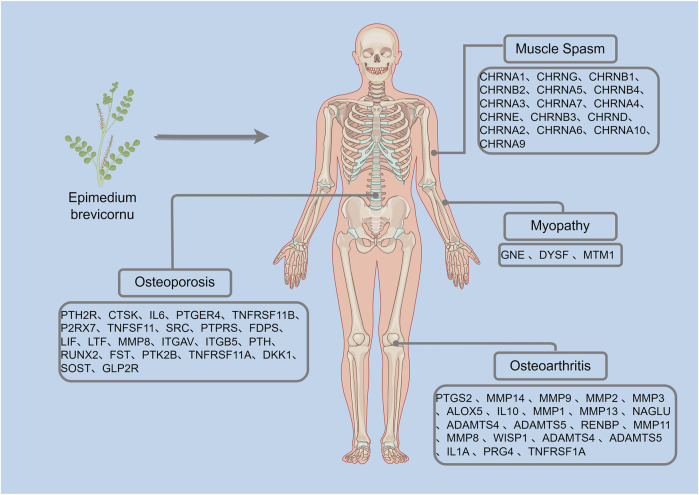
Therapeutic targets for orthopedic diseases shown in the TCMIP database (By Figdraw).

This study identified candidate targets of Epimedium active components through TCMIP database analysis based on skeletal system disease screening criteria. Using the “Disease-Target Association Analysis Module” of the database, therapeutic targets related to four pathological conditions “osteoarthritis, osteoporosis, muscle spasms, and myopathy” were screened. The therapeutic targets are shown in Figure 3.

**FIGURE 4:**
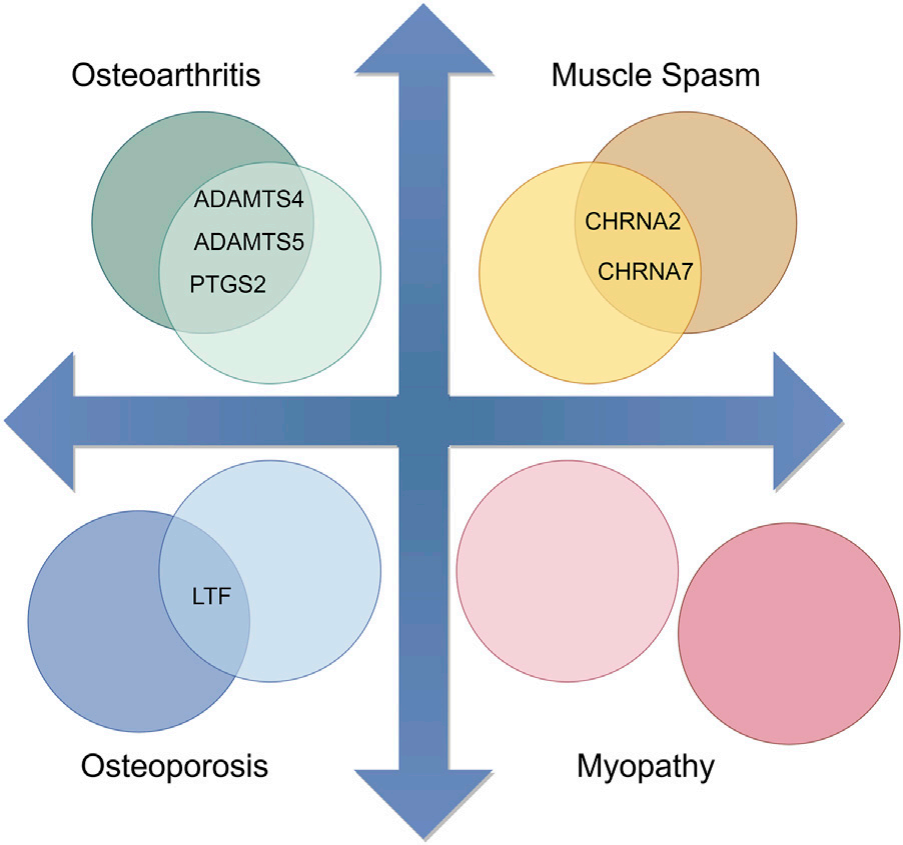
Overlap between Candidate Target Genes of major active components in Epimedium and Therapeutic Genes of Corresponding Diseases (By Figdraw).

From the model, we observed that except for Myopathy, there is a certain overlap between the therapeutic genes of the other three diseases and the candidate genes of the main active components of Epimedium, as shown in Figure 4. Therefore, we further utilized the Metascape tool to construct a network and perform KEGG enrichment analysis on the candidate target genes of the potentially therapeutic active substances in Epimedium and the therapeutic targets of the four related diseases identified through screening. This aims to analyze the potential therapeutic pathways of the main active components of Epimedium in treating these four types of diseases.

### 4.1 Possible pathway for epimedium in the treatment of osteoarthritis

**FIGURE 5:**
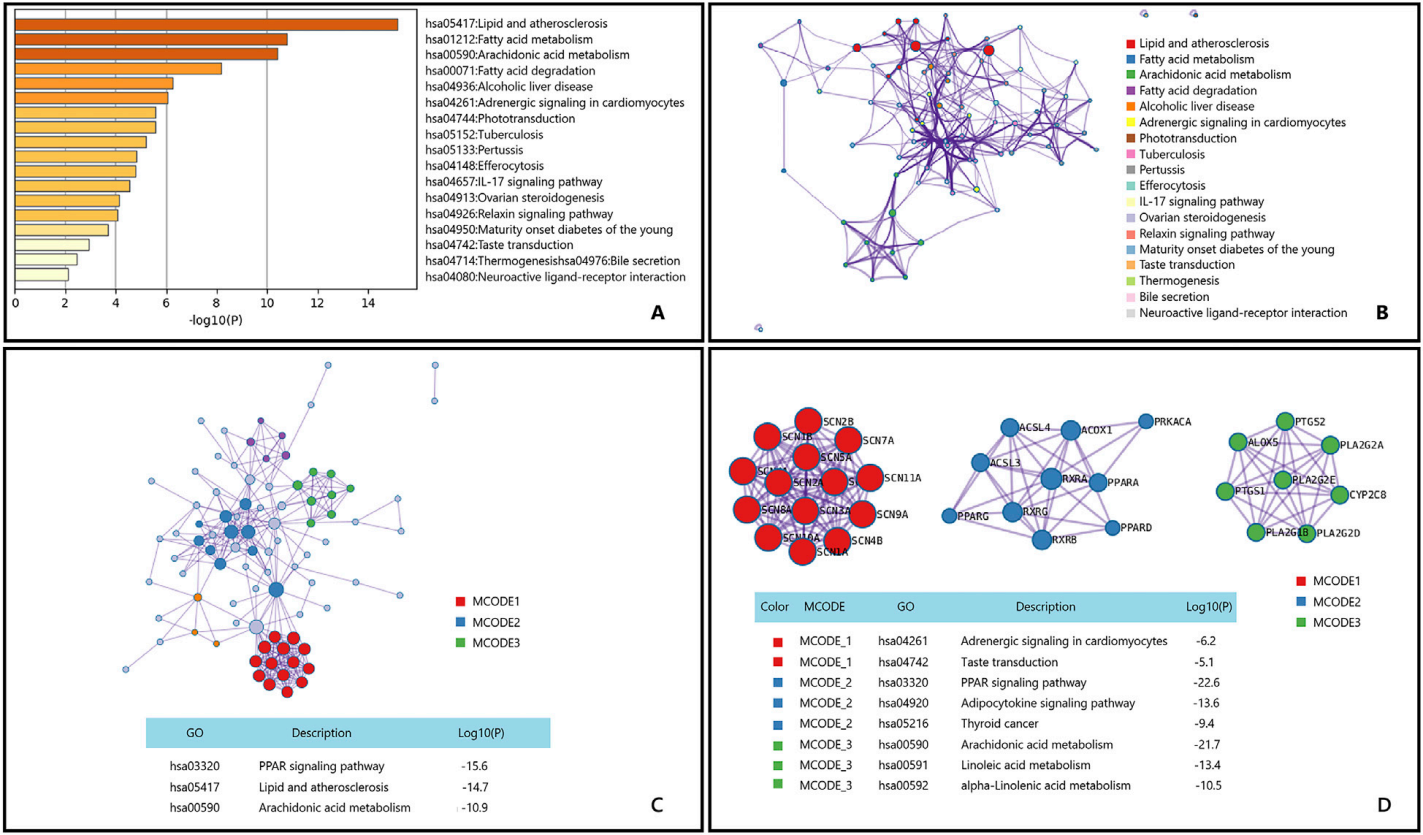
Gene List Analysis Report on the Main active ingredient of Epimedium to treat osteoarthritis **(A)** Bar graph of enriched terms across input gene lists, colored by p-values. **(B)** Network of enriched terms:colored by cluster ID, where nodes that share the same cluster ID are typically close to each other. **(C, D)** Protein-protein interaction network and MCODE componentsidentfedin the genelists. (The thre best-scoring tems by p-value have beenretained as thefunctional description of the corresponding components, shown in the tables undeneath corresponding network plots within Figure).

After performing KEGG enrichment analysis on the genes potentially involved in the treatment of osteoarthritis by the main active components of Epimedium using Metascape, the results are shown in Figure 5.

Osteoarthritis is a heterogeneous disease with an increasing incidence, mainly due to aging and obesity, leading to a significant global disease burden (Kloppenburg et al., 2025; Tang et al., 2025; Gelber, 2024). The Icariin,a role of active substances in non-alcoholic fatty liver disease (NAFLD) has been studied, showing that Icariin can improve hepatic fatty acid oxidation and inhibit lipid accumulation, which is closely related to their regulatory role in lipid metabolism (Hai et al., 2023). Additionally, the mechanism of Icariin in osteoarthritis has been preliminarily explored. Research indicates that Epimedium can alleviate chondrocyte apoptosis and thereby improve osteoarthritis symptoms by activating the SIRT-1-Nrf2-HO-1 signaling pathway (Liu YS. et al., 2024). Although this pathway is mainly associated with antioxidant stress and cytoprotection, it also indirectly participates in the regulation of lipid metabolism.These findings suggest that icariin may exert its therapeutic effects on osteoarthritis through multiple lipid metabolism-related pathways, including the Lipid and atherosclerosis pathway.

The Peroxisome Proliferator-Activated Receptor (PPAR) signaling pathway, renowned for its role in regulating lipid metabolism and inflammation, has been implicated in the pathogenesis of OA. Active components of Epimedium have demonstrated regulatory effects on human osteoarthritic fibroblast-like synoviocytes (OA-FLSs) *in vitro* studies (Pan et al., 2017). Moreover, Epimedium exerts its chondroprotective effects by inhibiting the expression of key enzymes in the MAPK signaling pathway (Zeng et al., 2017). These findings suggest that Epimedium may play a significant role in the treatment of osteoarthritis through the modulation of the PPAR signaling pathway and other related mechanisms. Research indicates that Epimedium can influence arachidonic acid metabolism by regulating the expression of PTGS1 and PTGS2 genes, thereby exerting its therapeutic effects (Liu et al., 2023). This mechanism may represent an important pathway through which Epimedium contributes to the treatment of osteoarthritis.

Based on the comprehensive KEGG enrichment analysis results, Epimedium may potentially treat osteoarthritis through the Lipid and atherosclerosis pathway, PPAR signaling pathway, and Arachidonic acid metabolism pathway.

### 4.2 Possible pathway for epimedium in the treatment of osteoporosis

**FIGURE 6:**
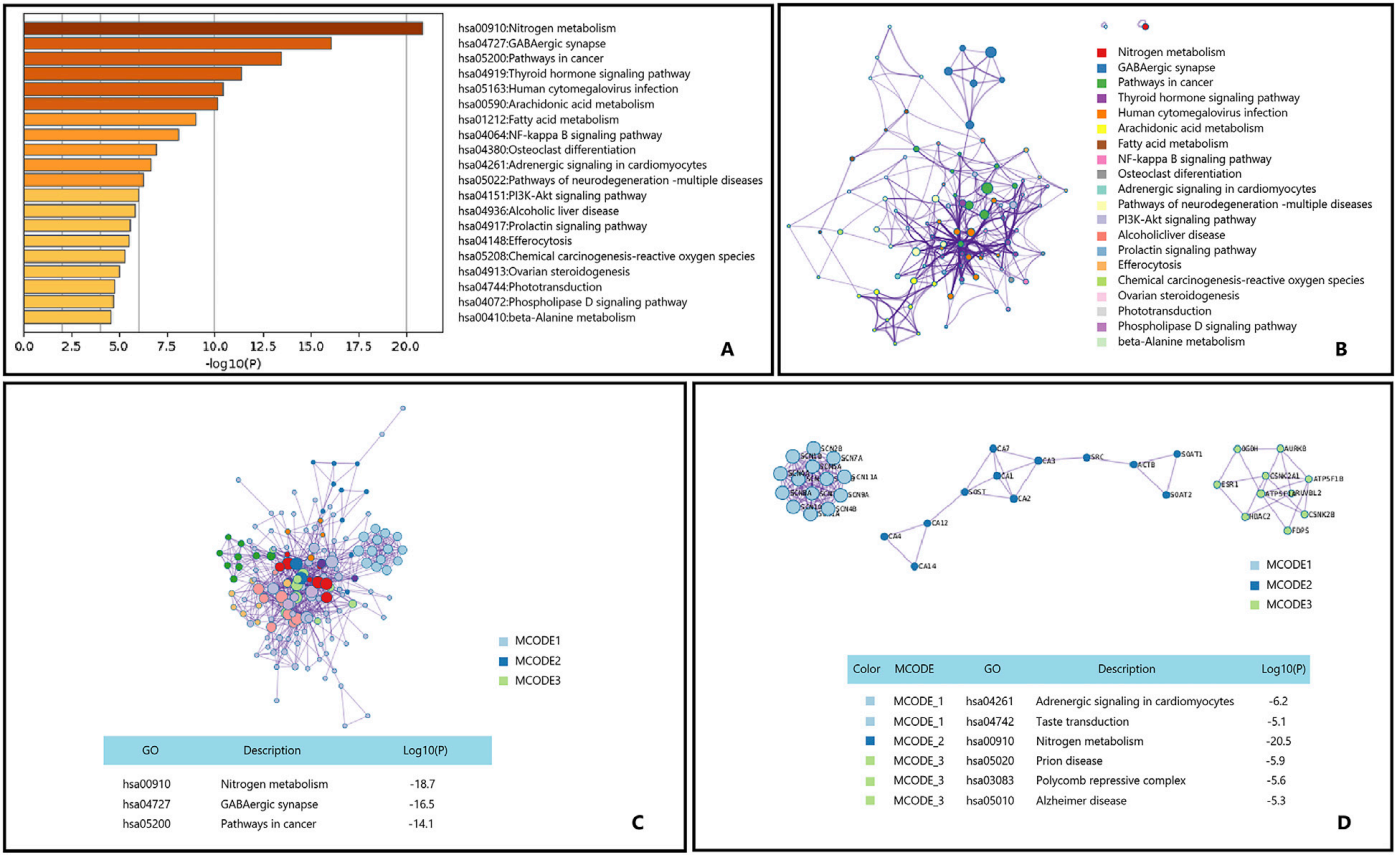
Gene List Analysis Report on the Main active ingredient of Epimedium to treat osteoporosis. **(A)** Bar graph of enriched terms across input gene lists, colored by p-values. **(B)** Network of enriched terms:colored by cluster ID, where nodes that share the same cluster ID are typically close to each other. **(C,D)** Protein-protein interaction network and MCODE componentsidentfedin the genelists. (The thre best-scoring tems by p-value have beenretained as thefunctional description of the corresponding components, shown in the tables undeneath corresponding network plots within Figure).

Osteoporosis is a prevalent metabolic bone disorder characterized by reduced bone density and deterioration of bone microarchitecture, leading to increased bone fragility and a higher risk of fractures (Ensrud and Crandall, 2018; Shapses, 2018). With the global trend of population aging, the incidence of osteoporosis is continuously rising, particularly among postmenopausal women (Ott, 2016; Michaëlsson and Aspenberg, 2016; Cheung et al., 2016). We utilized Metascape to perform KEGG enrichment analysis on the potential genes targeted by the main active components of Epimedium for the treatment of osteoporosis, and the results are illustrated in Figure 6.

Epimedium, a traditional Chinese medicine, has long been utilized in the treatment of osteoporosis. Research indicates that extracts from Epimedium can influence the levels of neuropeptides within the brain/spinal cord/bone axis, increasing the expression of neuropeptide Y (NPY) in the brain and receptors such as NPY1R in bone (Liu H. et al., 2018). This regulatory effect may have a positive impact on bone metabolism by affecting the nitrogen metabolism pathways. The GABAergic synapse pathway holds potential application value in the treatment of osteoporosis, as the regulation of GABA receptors may affect the proliferation and differentiation of bone cells, thereby exerting a beneficial effect on osteoporosis treatment (Gu et al., 2020; Wang et al., 2020; Yang et al., 2022). Although there is no direct evidence that Epimedium can treat osteoporosis through the GABAergic synapse pathway, our enrichment analysis results hint at this possibility. The cancer pathway also exhibits significant potential in the treatment of osteoporosis, with studies suggesting that it shares similar regulatory mechanisms in the treatment of osteoporosis (Gu et al., 2020; Amjadi-Moheb and Akhavan-Niaki, 2019; Meng et al., 2020; Zhu et al., 2019). For instance, the main active components of Epimedium leaves have been demonstrated to have a protective effect against osteoporosis by modulating the Wnt/β-catenin signaling pathway within the cancer pathway (Hu et al., 2017; Liu and Guo, 2020; Wang F. et al., 2016).

In summary, Epimedium may potentially treat osteoporosis through pathways such as Nitrogen metabolism, GABAergic synapse, and Pathways in cancer.

### 4.3 Possible pathway of epimedium in treating muscle spasms

**FIGURE 7:**
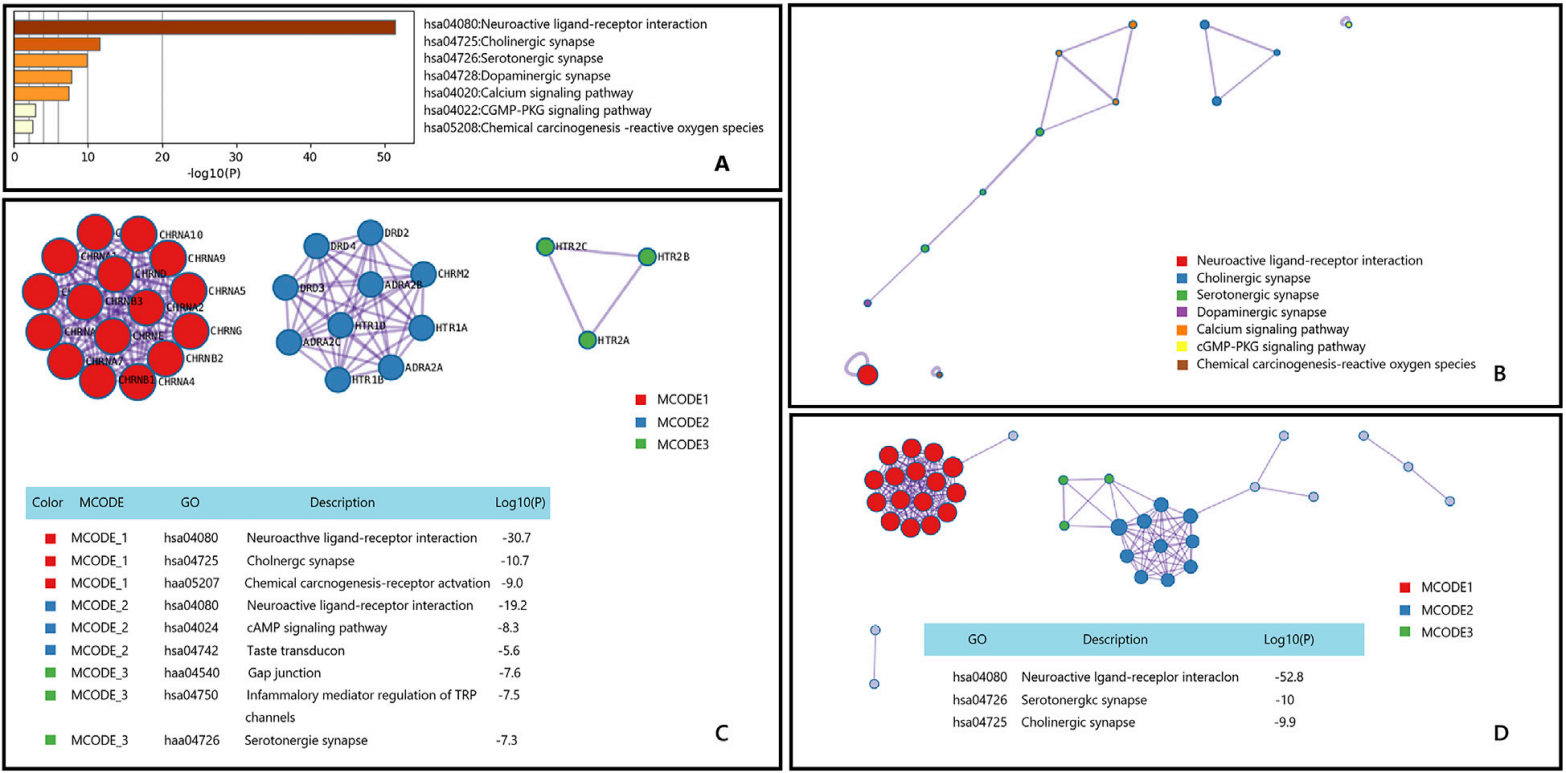
Gene List Analysis Report on the Main active ingredient of Epimedium to treat Muscle spasms. **(A)** Bar graph of enriched terms across input gene lists, colored by p-values. **(B)** Network of enriched terms:colored by cluster ID, where nodes that share the same cluster ID are typically close to each other. **(C,D)** Protein-protein interaction network and MCODE componentsidentfedin the genelists. (The thre best-scoring tems by p-value have beenretained as thefunctional description of the corresponding components, shown in the tables undeneath corresponding network plots within Figure).

Muscle spasms are a common symptom characterized by sudden, involuntary, and painful contraction of muscles. The pathophysiological mechanisms are not fully understood, but several hypotheses attempt to explain its occurrence. One hypothesis suggests that spasms are caused by changes in excitability of motor neurons (central origin), while another hypothesis suggests that they are caused by spontaneous discharges of motor neurons (peripheral origin) (Maughan and Shirreffs, 2019). Through Metascape’s KEGG enrichment analysis of genes associated with muscle spasms, we discovered that Epimedium may exert effects on muscle spasms via three signaling pathways closely related to nervous system function: Neuroactive ligand-receptor interaction, Serotonergic synapse, and Cholinergic synapse Figure 7.

Current research suggests that firstly, the active ingredients in Epimedium may exert their effects by affecting the levels of neurotransmitters in the central nervous system (Li et al., 2011). Secondly, Epimedium may exert its therapeutic effect by interacting with specific receptors (Wang et al., 2022). Additionally, Epimedium may also assist in alleviating muscle spasm through anti-inflammatory and antioxidant actions (Bäumer et al., 2014). Therefore, we posit that the active components in Epimedium may alleviate muscle spasms by modulating the release of neurotransmitters and the activation of receptors, which aligns with the results of our enrichment analysis.

#### 4.3.1 Possible pathway for epimedium in treating myopathy

**FIGURE 8:**
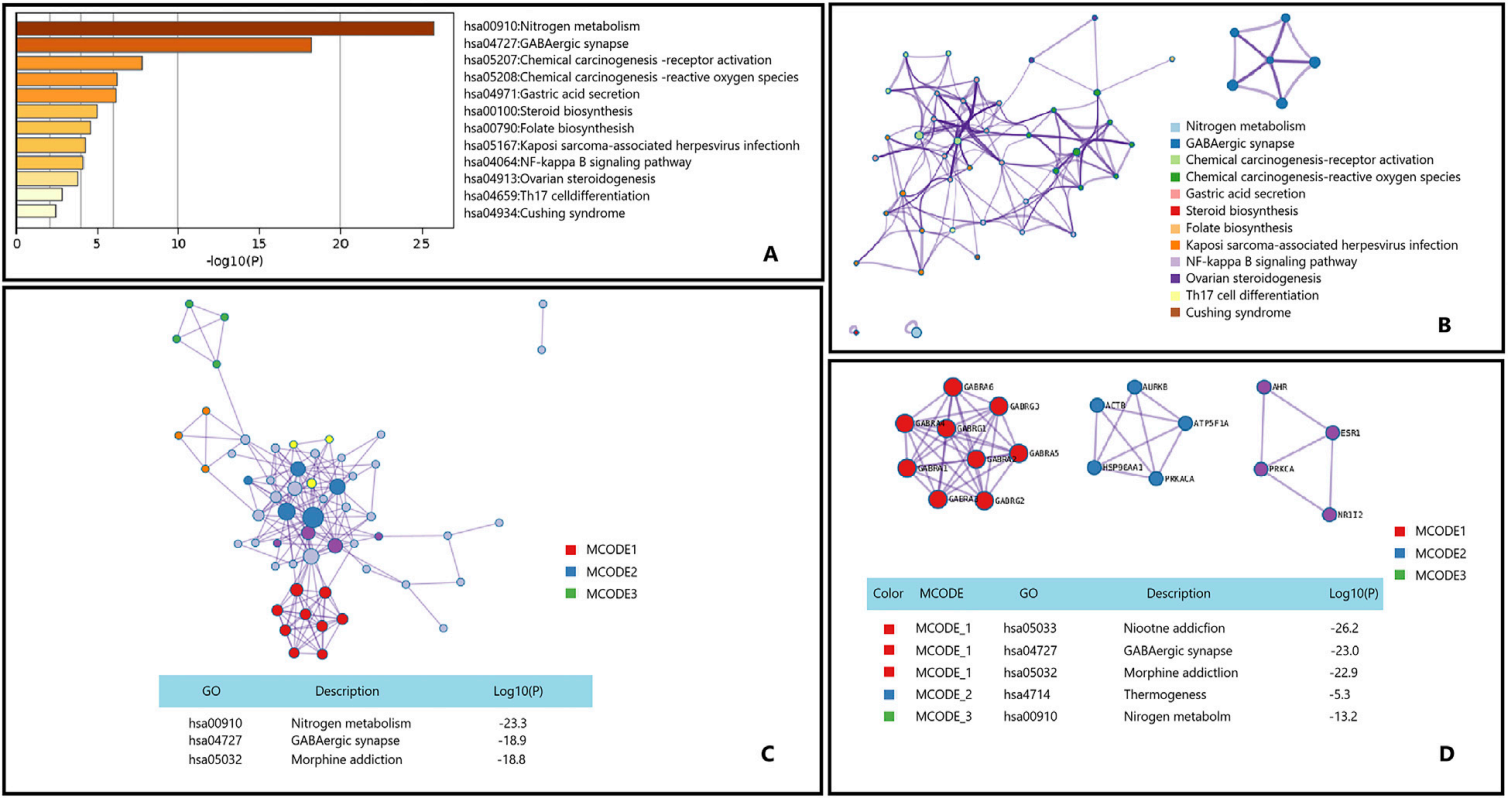
Gene List Analysis Report on the Main active ingredient of Epimedium to treat myopathy. **(A)** Bar graph of enriched terms across input gene lists, colored by p-values. **(B)** Network of enriched terms:colored by cluster ID, where nodes that share the same cluster ID are typically close to each other. **(C,D)** Protein-protein interaction network and MCODE componentsidentfedin the genelists. (The thre best-scoring tems by p-value have beenretained as thefunctional description of the corresponding components, shown in the tables undeneath corresponding network plots within Figure).

Myopathy refers to a group of diseases characterized primarily by muscle weakness due to dysfunction of muscle fibers. These conditions can be broadly categorized into several types, including congenital myopathies, inflammatory myopathies,and metabolic myopathies (Jungbluth et al., 2018; Maani et al., 2021; Ahmed et al., 2018). Epimedium and its active ingredients show significant therapeutic potential in muscle and nerve related diseases (Qian and Ke, 2020). Although there is no evidence that Epimedium can treat myopathies through Nitrogen metabolism and GABAergic synapse pathways, our analysis results may provide new perspectives on the application of Epimedium in the treatment of myopathy Figure 8.

## 5 Perspectives

Epimedium has long been widely used in traditional medicine, particularly in the treatment of orthopedic diseases, has demonstrated experimentally confirmed benefits for bone health through its bioactive components (Indran et al., 2016). However, several pharmacological limitations and safety concerns regarding its application warrant attention. Toxicological studies indicate that although aqueous Epimedium extracts exhibit low acute and chronic toxicity, they may induce hepatotoxicity in murine models (Song et al., 2024). Additionally, the relatively low bioactivity of icaritin (Gao and Zhang, 2022) significantly restricts its clinical translation potential. Future research should focus on systematic identification of active constituents, in-depth elucidation of pharmacological mechanisms, and optimization of drug delivery systems to enhance therapeutic efficacy while ensuring safety.

In this study,By analyzing the existing data in the TCMIP database, we found that among the 27 main active ingredients of Epimedium, 8 important ingredients have therapeutic effects on 11 types of Skeletal/Muscular related diseases.These diseases mainly include joint disorders (El-Shitany and Eid, 2019; Zhao et al., 2018), skeletal diseases (Liu et al., 2014; Wei CC. et al., 2016), muscle diseases (Lin et al., 2021), and pain related diseases (Zeng et al., 2017; Li et al., 2021), which almost comprehensively cover the spectrum of diseases related to bones, joints, muscles, and their associated tissues in terms of pathology.

Through the “Disease-Target Association Analysis Module,” we identified that Osteoarthritis, Osteoporosis, Muscle Spasm, and Myopathy have relatively clear therapeutic targets. Therefore, we conducted an enrichment analysis on these four types of diseases in conjunction with the candidate genes of Epimedium’s active components, predicting the possible pathways through which Epimedium may treat these diseases Table 4.

**TABLE 4:** Possible pathways of the main active ingredients of Epimedium in the treatment of orthopedic diseases.

Disease	Potential Mechanistic pathways	Hits
Osteoarthritis	Lipid and atherosclerosis	CALM1|CYP2C8|MMP1|MMP3|MMP9|PPARG|PPP3CA|PPP3R1|RXRA|RXRB|RXRG|TLR4|TNFRSF1A|VLDLR|LY96
	PPAR signaling pathway	ACOX1|ACSL3|ACSL4|MMP1|PPARA|PPARD|PPARG|RXRA|RXRB|RXRG|FADS2
	Arachidonic acid metabolism	ALOX5|CYP2C8|PLA2G1B|PLA2G2A|PTGS1|PTGS2|PLA2G2D|PLA2G2E
Osteoporosis	Nitrogen metabolism	CA1|CA2|CA3|CA4|CA5A|CA6|CA7|CA9|CA12|CA5B|CA14
	GABAergic synapse	ABAT|GABRA1|GABRA2|GABRA3|GABRA4|GABRA5|GABRA6|GABRG1|GABRG2|GABRG3|PRKACA|PRKCA|PRKCB|SRC
	Pathways in cancer	ABL1|AR|CALM1|CDK6|ESR1|ESR2|HDAC2|HSP90AA1|IL6|ITGAV|JAK1|PIM1|PPARD|PPARG|PRKACA|PRKCA|PRKCB|PTGER4|PTGS2|RXRA|RXRB|RXRG|NCOA1
Muscle Spasm	Neuroactive ligand-receptor interaction	ADRA2A|ADRA2B|ADRA2C|CHRM2|CHRNA1|CHRNA2|CHRNA3|CHRNA4|CHRNA5|CHRNA7|CHRNB1|CHRNB2|CHRNB3|CHRNB4|CHRND|CHRNE|CHRNG|DRD1|DRD2|DRD3|DRD4|DRD5|HTR1A|HTR1B|HTR1D|HTR2A|HTR2B|HTR2C|CHRNA6|CHRNA9|CHRNA10
	Serotonergic synapse	HTR1A|HTR1B|HTR1D|HTR2A|HTR2B|HTR2C|HTR3A
	Cholinergic synapse	ACHE|CHRM2|CHRNA3|CHRNA4|CHRNA7|CHRNB2|CHRNB4|CHRNA6
Myopathy	Nitrogen metabolism	CA1|CA2|CA3|CA4|CA5A|CA6|CA7|CA9|CA12|CA5B|CA14
	GABAergic synapse	GABRA1|GABRA2|GABRA3|GABRA4|GABRA5|GABRA6|GABRG1|GABRG2|GABRG3|PRKACA|PRKCA|PRKCB

The pathological mechanism of osteoarthritis are closely related to lipid metabolism, inflammatory responses and oxidative stress (Herrero-Beaumont et al., 2019; Tudorachi et al., 2021; Marchev et al., 2017; Mocanu et al., 2024; Hu et al., 2024), which may serve as potential targets for Epimedium in treating osteoarthritis. Involving an imbalance in bone metabolism, dysregulation of neuroendocrine functions,and potential cancer-related mechanisms (Liu C. et al., 2018; Zhang et al., 2022; Sharma et al., 2021), have been extensively studied in the context of Epimedium’s treatment of osteoporosis. In particular, the therapeutic effects of Epimedium on osteoporosis through cancer pathways such as the MAPK/ERK signaling pathway (Cao et al., 2017) and the NF-κBPathway (Yang et al., 2023) have been deeply investigated. The mechanisms underlying the treatment of muscle spasms may be related to abnormal signal transduction at the neuromuscular junction, involving the regulation of various neurotransmitter systems (Hezel et al., 2010; An et al., 2010; Colombo and Francolini, 2019). The pathological mechanisms of myopathy may be associated with abnormal protein metabolism and dysregulated neuromuscular signaling (Luzzi et al., 2023; Robb et al., 2011; Ignatieva et al., 2021), which could provide clues for researching the diagnosis and treatment of diseases with Epimedium.

In summary, Epimedium may demonstrate its potential therapeutic value in various diseases, including osteoarthritis, osteoporosis, muscle spasms, and myopathy by regulating key pathways such as lipid metabolism, inflammatory response, oxidative stress, bone metabolism balance, and neuromuscular signaling. These studies not only provide a molecular mechanism explanation for the pharmacological effects of Epimedium, but also lay a theoretical foundation for further development of precision treatment plans based on Epimedium. Although current research findings are primarily derived from bioinformatics analysis and preclinical experimental data, these discoveries offer a systematic theoretical framework for elucidating the pharmacological mechanisms of Epimedium and establish a critical foundation for subsequent in-depth molecular studies (such as precise modulation of key targets) and clinical translation (such as optimization of personalized dosing regimens). Future research should prioritize enhancing *in vitro* and *in vivo* experimental validation of Epimedium’s efficacy in treating osteoporosis, while further exploring the mechanisms of action of its active components within specific pathological microenvironments, aiming to achieve a transformative leap from traditional applications to evidence-based medicine.

## Abbreviations

Yin Yang Huo, Epimedium brevicornu; ETCM, The Encyclopedia of Traditional Chinese Medicine; YaTCM, Yet another Traditional Chinese Medicine; TCMIP, Integrative Pharmacology-based Research Platform of Traditional Chinese Medicine; BMP, Bone morphogenetic protein; NF-κB, Nuclear factor kappa B; RA, Rheumatoid arthritis; OPG, Osteoprotegerin; RANKL, Receptor activator of nuclear factor-κB ligand; OA, Osteoarthritis; NAFLD, Non-alcoholic fatty liver disease; PPAR, Peroxisome Proliferator-Activated Receptor; OA-FLSs, Osteoarthritic fibroblast-like synoviocytes; NPY, Neuropeptide Y.
